# Assessment of the Diagnostic Accuracy of Biparametric Magnetic Resonance Imaging for Prostate Cancer in Biopsy-Naive Men: The Biparametric MRI for Detection of Prostate Cancer (BIDOC) Study

**DOI:** 10.1001/jamanetworkopen.2018.0219

**Published:** 2018-06-08

**Authors:** Lars Boesen, Nis Nørgaard, Vibeke Løgager, Ingegerd Balslev, Rasmus Bisbjerg, Karen-Cecilie Thestrup, Mads D. Winther, Henrik Jakobsen, Henrik S. Thomsen

**Affiliations:** 1 Department of Urology, Herlev Gentofte University Hospital, Herlev, Denmark; 2 Department of Radiology, Herlev Gentofte University Hospital, Herlev, Denmark; 3 Department of Pathology, Herlev Gentofte University Hospital, Herlev, Denmark

## Abstract

**Importance:**

Multiparametric magnetic resonance imaging (MRI) enhances detection and risk stratification for significant prostate cancer but is time-consuming (approximately 40 minutes) and expensive. Rapid and simpler (approximately 15-minute) biparametric MRI (bpMRI) using fewer scan sequences could be implemented as a prostate MRI triage test on a larger scale before performing biopsies.

**Objectives:**

To assess the diagnostic accuracy and negative predictive value (NPV) of a novel bpMRI method in biopsy-naive men in detecting and ruling out significant prostate cancer in confirmatory biopsies.

**Design, Setting, and Participants:**

A single-institutional, paired, prospective cohort study of biopsy-naive men with clinical suspicion of prostate cancer from November 1, 2015, to June 15, 2017.

**Interventions:**

All patients underwent bpMRI (T2-weighted and diffusion-weighted imaging) followed by standard transrectal ultrasound-guided biopsies (all men) and targeted biopsies of men with suspicious bpMRI findings.

**Main Outcomes and Measures:**

Suspicion grades of bpMRI, biopsy results, and NPV of bpMRI were evaluated for detection of or ruling out significant prostate cancer (Gleason score ≥4 + 3 or maximum cancerous core length >50% for Gleason score 3 + 4). We compared the diagnostic performance of standard biopsies in all men vs standard plus targeted (combined) biopsies restricted to men with suspicious bpMRI findings. The reference standard was combined biopsy results from all men.

**Results:**

A total of 1020 men were enrolled, with a median age of 67 years (interquartile range, 61-71 years) and a median prostate-specific antigen level of 8.0 ng/mL (interquartile range, 5.7-13.0 ng/mL). Combined biopsies detected any and significant prostate cancer in 655 of 1020 men (64%) and 404 of 1020 men (40%), respectively. Restricting combined biopsies to men with suspicious bpMRI findings meant 305 of 1020 men (30%) with low-suspicious bpMRIs could avoid prostate biopsies (biopsy in 715 men with suspicious bpMRIs vs all 1020 men who required standard biopsies [70%]; *P* < .001). Significant prostate cancer diagnoses were improved by 11% (396 vs 351 men; *P* < .001), and insignificant prostate cancer diagnoses were reduced by 40% (173 vs 288 men; *P* < .001) compared with our current diagnostic standard, standard biopsies alone in all men. The NPV of bpMRI findings in ruling out significant prostate cancer was 97% (95% CI, 95%-99%).

**Conclusions and Relevance:**

Low-suspicion bpMRI has a high NPV in ruling out significant prostate cancer in biopsy-naive men. Using a simple and rapid bpMRI method as a triage test seems to improve risk stratification and may be used to exclude aggressive disease and avoid unnecessary biopsies with its inherent risks. Future studies are needed to fully explore its role in clinical prostate cancer management.

## Key Points

**Question:**

What are the diagnostic accuracy and negative predictive value of novel biparametric magnetic resonance imaging (MRI) in biopsy-naive men in detecting and ruling out significant prostate cancer?

**Findings:**

In this cohort study of 1020 men who underwent both biparametric targeted and standard transrectal ultrasound-guided biopsies, low-suspicion biparametric MRI had a high negative predictive value (97%) in ruling out significant prostate cancer on confirmatory biopsies.

**Meaning:**

The biparametric MRI used as a triage test in this study was associated with improved prostate cancer risk stratification and may be used to exclude aggressive disease and avoid unnecessary biopsies in 30% of men with clinical suspicion of prostate cancer, although further studies are needed to fully explore this new diagnostic approach.

## Teaser

This study assesses the diagnostic accuracy and negative predictive value of biparametric magnetic resonance imaging (bpMRI) methods for detecting clinically significant prostate cancer in biopsy-naive men.

## Introduction

Standard diagnostic transrectal ultrasonography (TRUS)–guided biopsies are offered to men with clinical suspicion of prostate cancer due to elevated prostate-specific antigen (PSA) levels and/or abnormal digital rectal examination results. However, men without prostate cancer undergo unnecessary biopsies because elevated PSA is not cancer specific. Given the high false-positive rate of PSA, its use for screening purposes is controversial and an area of continuous debate within the medical and urological communities.^1^ As standard biopsies are prone to sampling errors because of difficulties in prostate cancer target identification on TRUS, clinically significant prostate cancer may be missed and insignificant prostate cancer detected by the random untargeted sampling, potentially leading to overdetection and overtreatment.^2^ In addition, biopsies are invasive and may lead to patient anxiety and morbidity.^3^ These limitations have highlighted the need for better diagnostic tools, such as risk calculators, biomarkers, or imaging techniques,^4^ to improve selection of men with increased risk of significant prostate cancer who require diagnostic biopsies and subsequent treatment from the proportion of men with either a benign condition or an insignificant prostate cancer that can be managed with expectancy. However, risk calculators are highly influenced by the population studied and newer biomarkers can be costly, may be limited by availability, and have not yet been proven to have the desired level of accuracy in biopsy-naive men with prostate cancer.^5,6^ Accurate methods that improve detection of significant prostate cancer while minimizing overdetection and unnecessary biopsies by reducing the number of false-positive results are highly warranted. Growing evidence supports the use of multiparametric magnetic resonance imaging (mpMRI) to solve this problem.^7,8^ Magnetic resonance imaging–guided biopsies (targeted biopsies) can be targeted toward the most aggressive part of suspicious lesions detected by mpMRI, improving the detection of significant prostate cancer compared with standard biopsies alone.^9,10,11,12^ Conversely, low-suspicion mpMRI may noninvasively exclude the presence of aggressive disease.^13^ Accordingly, mpMRI could potentially be used as a triage test to identify biopsy-naive men with clinical suspicion of prostate cancer who might safely avoid unnecessary biopsies.

However, guidelines on prostate MRI^14,15,16^ recommend a full mpMRI prostate examination that includes several anatomical and functional scan sequences as well as intravenous contrast media. This is time-consuming (approximately 40 minutes), expensive, and might be difficult to implement on a large scale. Over time it has become evident that contrast-enhanced imaging and multiple imaging planes often do little to improve the overall clinical picture, especially in the detection and localization of significant prostate cancer. In contrast, a rapid and simple biparametric MRI (bpMRI) method that uses fewer scan sequences and no intravenous contrast media might decrease image acquisition time (approximately 15 minutes) and costs, while retaining sufficient diagnostic accuracy to detect and rule out significant prostate cancer in biopsy-naive men. Such a bpMRI protocol could provide a basis for a prostate MRI triage test prior to biopsy. Consequently, this prospective study assesses the diagnostic accuracy of bpMRI in detecting and ruling out significant prostate cancer in biopsy-naive men with clinical suspicion of prostate cancer. We evaluated the clinical significance of detected cancers and assessed whether bpMRI could be used as a triage test to improve the diagnosis of significant prostate cancer and identify patients who could safely avoid unnecessary biopsies.

## Methods

This Biparametric MRI for Detection of Prostate Cancer (BIDOC) study is a prospective, single-institution, paired-cohort study. It was approved by the Local Committee for Health Research Ethics and the Danish Data Protection Agency. Participants provided written informed consent and were enrolled from November 1, 2015, to June 15, 2017. This study conformed to the Standards of Reporting for MRI-Targeted Biopsy Studies consortium criteria for MRI biopsy studies^17^ and adhered to the Standards for Reporting of Diagnostic Accuracy (STARD) reporting guideline criteria.^18^ The study inclusion criteria required all men to have clinical suspicion of prostate cancer (PSA ≥4 ng/mL [to convert to micrograms per liter, multiply by 1.0] and/or abnormal digital rectal examination results) that warranted a diagnostic prostate biopsy. The exclusion criteria were prior prostate biopsies, evidence of acute urinary tract infections, acute prostatitis, general contraindications for MRI (eg, claustrophobia, a pacemaker, metal implants), and prior hip replacement surgery or other metallic implants in the pelvic area.

### Outcome Measures

The primary end points were the diagnostic accuracy and negative predictive value (NPV) of low-suspicion bpMRI findings in ruling out significant prostate cancer in confirmatory biopsies from biopsy-naive men. Secondary end points included the overall prostate cancer detection rate and detection rates of significant prostate cancer and insignificant prostate cancer stratified by biopsy technique. We also evaluated the clinical value of using bpMRI as a triage test prior to biopsies and estimated the proportion of men who could safely avoid unnecessary biopsies based on low-suspicion bpMRI findings.

### bpMRI (Index Test) and Image Analysis

Prior to biopsies, bpMRI was performed using a 3-T MRI magnet (Philips Healthcare) with a pelvic-phased-array coil (Philips Healthcare) positioned over the pelvis. The bpMRI protocol included axial T2-weighted and diffusion-weighted images (*b *values: 0, 100, 800, and 2000) with reconstructions of the corresponding apparent diffusion coefficient map, because these 2 parameters are the dominant sequences for prostate cancer lesion detection on mpMRI.^15^ A sagittal T2-weighted luxury scout image supported the axial sequences for MRI/TRUS image fusion. The overall bpMRI image acquisition time was approximately 15 minutes. Imaging parameters are listed in eTable 1 in the Supplement.

All bpMRI images were reviewed by the same prostate MRI physician (>5 years of experience) blinded to clinical findings. Suspicious lesions were scored on a 5-point scale according the Prostate Imaging Reporting and Data System version 2 (PI-RADSv2) criteria.^15^ However, as the bpMRI protocol does not include dynamic contrast-enhanced imaging, scoring of lesions in the peripheral zone relied solely on diffusion-weighted image findings (dominant sequence), and an equivocal score of 3 was not potentially upgraded to a score of 4 due to lack of positive dynamic contrast-enhanced findings. All patients were graded overall using this modified PI-RADS score according to their likelihood of having significant prostate cancer (1, highly unlikely; 2, unlikely; 3, equivocal; 4, likely; and 5, highly likely). A modified PI-RADS suspicion score of 2 or lower was perceived as a low-suspicion or negative bpMRI scan result. Patients with no suspicious lesions were assigned an overall modified PI-RADS score of 1.

### Standard and Targeted Biopsies

Initially, all patients underwent systematic standard biopsies (10-core extended sextant biopsy scheme) according to guidelines.^19^ Any suspicious lesion detected by TRUS was sampled as part of the standard biopsy scheme. Standard biopsies were immediately followed by additional targeted biopsies of any bpMRI suspicious lesions (modified PI-RADS≥3; 1-2 cores/lesion) using 1 of 2 rigid MRI/TRUS image-fusion systems: HI-RVS system (Hitachi; n = 877) and Uro-Nav system (Invivo; n = 143) for men biopsied. All prostate biopsies were potted separately and obtained using an end-fire biopsy technique by 1 of 2 operators with extensive experience in performing standard biopsies and reasonable experience in software-based image fusion for targeted biopsies (4 years and 1 year). Performance and analysis of standard biopsies and bpMRI were blinded with respect to each other.

### Histopathological Evaluation and Cancer Significance

All biopsy samples were reviewed by the same genitourinary pathologist (>15 years of experience). For each prostate cancer-positive biopsy core, the location, Gleason score (GS) based on the International Society of Urological Pathology 2005 consensus,^20^ and percentage of cancerous tissue per core were determined. In addition, patients were allocated using the International Society of Urological Pathology 2014 consensus Gleason-grade groups^21^ based on the GS scoring criteria.^20^ The primary definition of significant prostate cancer included both cancer GS grade and volume, and significant prostate cancer was defined as any core with high-grade prostate cancer (GS ≥7 [4 + 3]) or maximum cancerous core length greater than 50% of GS 7 (3 + 4) prostate cancer. Other definitions of significant prostate cancer were additionally assessed.

### Statistical Analysis

Patient characteristics were stratified by biopsy results and reported using descriptive statistics. Continuous variables (eg, age, PSA level, PSA density, and prostate volume) were compared using the Wilcoxon rank sum test. Fisher exact test was used to compare the clinical tumor stage determined by digital rectal examination pooled in nonpalpable and palpable tumor groups. Prebiopsy bpMRI suspicion (modified PI-RADS) scores were compared with biopsy results using a χ^2^ analysis to determine the association between bpMRI suspicion and positive biopsy findings. We compared the diagnostic performances of the following clinical strategies: (1) standard biopsies in all men, (2) standard plus targeted (combined) biopsies restricted to men with suspicious bpMRIs, and (3) combined biopsies in all men, which served as reference standard. Any patient with significant prostate cancer in either standard or targeted biopsies was classified as having significant prostate cancer on combined biopsies. A McNemar test was used to compare prostate cancer detection rates between biopsy strategies in 2 × 2 contingency tables. The sensitivity and NPV for detecting and ruling out any prostate cancer and significant prostate cancer comparing standard biopsies in all men vs combined biopsies restricted to men with suspicious bpMRIs were calculated to assess our primary outcome measures. Furthermore, the clinical value of the biopsy strategies comparing benefits (significant prostate cancer detection) and harms (unnecessary biopsies) were evaluated using net benefit and decision curve analyses. All anayses were 2-tailed and a *P *value of less than .05 was considered significant. Statistical analyses were performed using SPSS statistical software version 22.0 (SPSS Inc).

## Results

**Figure 1.**
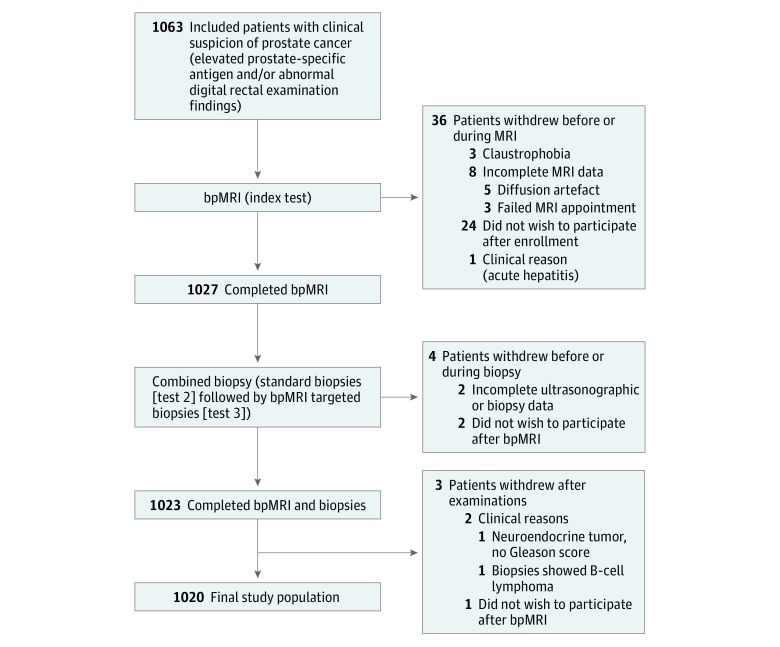
Flowchart of the Study Population A total of 1063 men were included. However, 43 were excluded for various reasons. The final study population consisted of 1020 men who completed all examinations. MRI indicates magnetic resonance imaging; bpMRI, biparametric MRI.

A total of 1063 men were prospectively enrolled and 43 were excluded for various reasons (Figure 1). The final study population consisted of 1020 men with a median age of 67 years (interquartile range, 61-71 years) and a median PSA level of 8.0 ng/mL (interquartile range, 5.7-13.0 ng/mL). The patients’ demographic data and baseline characteristics are listed in Table 1. Overall, prostate cancer was detected in 655 of 1020 men (64%), and 404 of 1020 men (40%) had significant prostate cancer according to the primary definition. Standard biopsies detected prostate cancer and significant prostate cancer in 639 of 1020 men (63%) and 351 of 1020 men (34%), respectively, with 402 of 639 men (63%) having lower-grade prostate cancer (Gleason-grade group 1 or 2). We found a lower NPV for any prostate cancer (72%) for a modified PI-RADS score of 3 or higher, but a higher NPV for significant prostate cancer (97%). Targeted biopsies were performed for 715 of 1020 men (70%) with suspicious bpMRIs (modified PI-RADS≥3) and detected prostate cancer and significant prostate cancer in 478 of 715 men (67%) and 338 of 715 men (47%), respectively. Patients with low-suspicion bpMRIs (305 men [30%]) did not have targeted biopsies. Of these, standard biopsies detected prostate cancer in 86 of 305 men (28% [8% of the entire cohort]) stratified into 78 of 305 men (26% [8% of the entire cohort]) with insignificant prostate cancer and 8 of 305 men (3% [0.8% of the entire cohort]) with significant prostate cancer (Table 2).

**Table 1.** Patient Characteristics

Clinical Characteristic	Prostate Cancer Negative (n = 365)	Prostate Cancer Positive (n = 655)^a^	*P* Value	Total (N = 1020)
Age, median (IQR), y	64 (59-69)	68 (62-72)	<.001	67 (61-71)
PSA, median (IQR), ng/mL	6.4 (5.2-8.9)	9.2 (6.1-19.9)	.03	8.0 (5.7-13.0)
Prostate volume, median (IQR), cm^3^	65 (49-88)	47 (36-61)	<.001	53 (40-72)
PSA density, median (IQR), ng/mL/cm^3^	0.10 (0.07-0.14)	0.20 (0.12-0.43)	<.001	0.15 (0.10-0.27)
Time from bpMRI to biopsy, median (IQR), d	7 (4-11)	7 (5-9)	.44	7 (7-9)
cT_DRE_ stage, No. (%)				
Nonpalpable tumor			<.001^b^	
Tx	106 (29)	69 (11)		175 (17)
T1c	208 (57)	260 (40)		468 (46)
Palpable tumor				
T2	46 (13)	199 (30)		245 (24)
T3	5 (1)	120 (18)		125 (12)
T4	0	7 (1)		7 (1)

Abbreviations: bpMRI, biparametric magnetic resonance imaging; cT_DRE_, tumor stage determined by digital rectal examination; IQR, interquartile range; PSA, prostate-specific antigen.

SI conversion factor: To convert PSA to micrograms per liter, multiply by 1.0.

^a^ Based on biopsy results of combined biopsies in all men.

^b^ A Fisher exact test was used to compare the cT_DRE_ stage pooled in nonpalpable and palpable tumor groups.

**Table 2.** Comparison of bpMRI Suspicion Scores With Biopsy Gleason Scores and Grade Groups^a^

bpMRI Modified PI-RADS^b^	Combined Biopsies, No.^c^							
	No Prostate Cancer	Insignificant Prostate Cancer		Significant Prostate Cancer				Total
		GS 6, GGG 1	GS 3 + 4, GGG 2, MCCL ≤50%	GS 3 + 4, GGG 2, MCCL >50%	GS 4 + 3, GGG 3	GS 8, GGG 4	GS 9 to 10, GGG 5	
1	123	44	8	1	2	2	1	181
2	97	20	5	0	2	0	0	124
3	64	38	11	7	6	2	2	130
4	47	38	29	29	22	15	7	187
5	35	39	18	74	76	64	92	398
Total	366	179	71	111	108	83	102	1020

Abbreviations: bpMRI, biparametric magnetic resonance imaging; GGG, Gleason-grade group; GS, Gleason score; MCCL, maximum cancer-core length; PI-RADS, Prostate Imaging Reporting and Data System.

^a^ Biopsy results of all patients were stratified by GS/GGG and bpMRI modified PI-RADS. Gleason-grade group 2 (GS 3 + 4) was subdivided into 2 groups (MCCL ≤50% and MCCL >50%).

^b^ A bpMRI modified PI-RADS score of 1 or 2 indicates low-suspicion or negative bpMRI findings; a bpMRI modified PI-RADS score of 3 or 5 indicates suspicious bpMRI findings.

^c^ Patients with a modified PI-RADS score of 1 or 2 only underwent standard transrectal ultrasound-guided biopsies. Combined biopsies are standard plus targeted.

**Figure 2.**
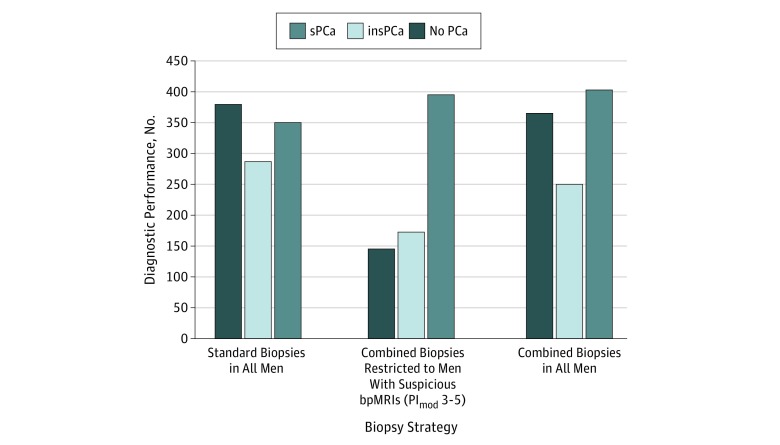
Comparison of the Diagnostic Performances of Biopsy Strategies The diagnostic performance consisted of standard biopsies in all men (N = 1020), combined (standard plus targeted) biopsies restricted to men with suspicious biparametric magnetic resonance imaging (bpMRI) findings (n = 715), and combined biopsies in all men (reference standard) (N = 1020). Biopsy results were stratified by cancer significance (primary definition). insPCa indicates insignificant prostate cancer; PCa, prostate cancer; PI_mod_, modified Prostate Imaging Reporting and Data System score; and sPCa, significant PCa.

The bpMRI modified PI-RADS suspicion scores were associated with the biopsy results (*P* < .001) (eFigure 1 in the Supplement). The diagnostic yield of significant prostate cancer increased at higher modified PI-RADS scores, and there was a significantly lower significant prostate cancer detection rate in men with low-suspicion bpMRI findings compared with men who had highly suspicious (modified PI-RADS 4-5) bpMRI findings (8 of 305 [3%, or 0.8% of the entire cohort] vs 379 of 585 [65%, or 57% of the entire cohort]; *P* < .001). The diagnostic performances of standard and combined biopsies are shown in Figure 2. The value of using bpMRI as a diagnostic triage test to identify men most suitable for prostate biopsies—to identify significant prostate cancers and avoid unnecessary biopsies—was assessed by comparing standard biopsies in all men vs combined biopsies restricted to men with suspicious bpMRIs (Table 3). Restricting combined biopsies to men with suspicious bpMRI findings meant 305 of 1020 men (30%) with low-suspicious bpMRIs could avoid primary prostate biopsies (biopsy 715 men with suspicious bpMRIs vs all 1020 men who required standard biopsies [70%]; *P* < .001). Significant prostate cancer diagnoses were improved by 11% (4% absolute improvement; 396 vs 351 men; *P* < .001), and insignificant prostate cancer diagnoses were reduced by 40% (11% absolute reduction; 173 vs 288 men; *P* < .001) using fewer biopsy cores compared with standard biopsies alone. The NPV of bpMRI findings in ruling out significant cancer was 97% (95% CI, 95%-99%). Standard biopsies detected significant prostate cancer in 8 men with modified PI-RADS scores of 2 or lower (eTable 2 in the Supplement). Other definitions of significant prostate cancer were also used to evaluate the 2 biopsy strategies (Table 3). Although the prevalence of significant prostate cancers changed when other definitions were used, the reduction in diagnoses in men with insignificant prostate cancer when bpMRI was used as a triage test did not change markedly. However, for the tertiary definition of significant prostate cancer (GS ≥3 + 4), the detection rate for the comparison between standard and combined biopsies did not reach the level of statistical significance (McNemar test, *P* = .11). Sensitivities, NPVs, and net benefit with decision curve analyses are compared in Table 4 and eFigure 2 in the Supplement. Furthermore, restricting combined biopsies to men with suspicious bpMRIs compared with performing combined biopsies in all men reduced overdiagnosis of insignificant prostate cancer by 31% (n = 77; 173 vs 250 men).

**Table 3.** Comparison of Biopsy Strategy^a^

Significant Prostate Cancer Definition	Biopsies, No. (% [95% CI])^b^		Difference, % (95% CI)		*P* Value, McNemar Test
	Standard (All Men)	Combined (modified PI-RADS 3-5)	Absolute	Relative	
Men with biopsy performed	1020 (100 [99 to 100])	715 (70 [67 to 73])	–30 (–33 to –27)	–30 (–33 to –27)	<.001
Biopsy cores, No.	9268	7339^c^	–21 (–22 to –20)	–21 (–22 to –20)	<.001
No prostate cancer on biopsy	381 (37 [34 to 40])	146 (14 [12 to 17])	–23 (–27 to –19)	–62 (–68 to –55)	<.001
Primary definition of significant prostate cancer^d^					
Insignificant prostate cancer	288 (28 [26 to 31])	173 (17 [15 to 19])	–11 (–15 to –8)	–40 (–49 to –29)	<.001
Significant prostate cancer	351 (34 [32 to 37])	396 (39 [36 to 42])	4 (0.2 to 9)	11 (0.6 to 21)	<.001
Secondary definition of significant prostate cancer^e^					
Insignificant prostate cancer	262 (25 [23 to 29])	145 (14 [12 to 17])	–11 (–15 to –8)	–45 (–54 to –34)	<.001
Significant prostate cancer	377 (37 [34 to 40])	424 (42 [39 to 45])	5 (0.4 to 9)	11 (0.9 to 21)	<.001
Tertiary definition of significant prostate cancer^f^					
Insignificant prostate cancer	198 (19 [17 to 22])	115 (11 [9 to 13])	–8 (–11 to –5)	–42 (–53 to –28)	<.001
Significant prostate cancer	441 (43 [40 to 46])	454 (45 [41 to 48])	1 ( to 3 to 6)	3 (−7 to 12)	.11

Abbreviation: PI-RADS, Prostate Imaging Reporting and Data System.

^a^ Comparison of the diagnostic strategies of standard biopsies in all men vs combined (standard plus targeted) biopsies restricted to men with suspicious biparametric magnetic resonance imaging findings (modified PI-RADS score 3-5) using different definitions of significant prostate cancer.

^b^ The total number of patients (N = 1020) was used as the denominator for calculating all percentages.

^c^ Includes 6231 standard biopsies and 1108 targeted biopsies.

^d^ Gleason score of 4 + 3 or greater or maximum cancer-core length greater than 50% with a Gleason score of 3 + 4. The prevalence was 404 men (40% [95% CI, 37%-43%]).

^e^ Gleason score of 4 + 3 or greater or maximum cancer-core length greater than 50% with any PCa. The prevalence was 436 men (43% [95% CI, 40%-46%]).

^f^ Gleason score of 3 + 4 or greater. The prevalence was 475 men (47% [95% CI, 44%-50%]).

**Table 4.** Comparison of Sensitivities and NPVs

Prostate Cancer Definition^a^	% (95% CI)		
	bpMRI Modified PI-RADS 3-5 (n = 715)^b^	Standard Biopsies, All Men (N = 1020)	Combined Biopsies, Modified PI-RADS 3-5 (n = 715)
Any prostate cancer^c^			
Sensitivity	86 (84-89)	98 (96-99)	86 (84-89)
NPV	72 (67-76)	96 (93-97)	81 (78-84)
Significant prostate cancer, primary definition^d^			
Sensitivity	98 (96-99)	87 (83-90)	98 (96-99)
NPV	97 (95-99)	92 (90-94)	99 (97-99)
Significant prostate cancer, secondary definition^e^			
Sensitivity	97 (95-99)	86 (83-90)	97 (95-99)
NPV	96 (93-98)	91 (89-93)	98 (97-99)
Significant prostate cancer, tertiary definition^f^			
Sensitivity	96 (93-97)	93 (90-95)	96 (93-97)
NPV	93 (90-95)	94 (92-96)	96 (94-98)

Abbreviations: bpMRI, biparametric magnetic resonance imaging; NPV, negative predictive value; PI-RADS, Prostate Imaging Reporting and Data System.

^a^ The sensitivities and NPVs for detecting and ruling out any PCa and sPCa are shown for bpMRI alone and for the 2 diagnostic strategies (1) standard biopsies in all men and (2) combined biopsies (standard plus targeted) restricted to men with suspicious bpMRIs (modified PI-RADS 3-5) using various definitions of significant PCa. Overall detection rates of PCa and sPCa in combined standard transrectal ultrasonography-guided biopsies and bpMRI targeted biopsies of all patients was used as the reference standard.

^b^ The bpMRI score was dichotomized by low-suspicion or negative bpMRI findings (modified PI-RADS 1-2) and suspicious bpMRI findings (modified PI-RADS 3-5).

^c^ Prevalence was 655 men (64% [95% CI, 61%-67%]).

^d^ Gleason score of 4 + 3 or greater or maximum cancer-core length greater than 50% with a Gleason score of 3 + 4. The prevalence was 404 men (40% [95% CI, 37%-43%]).

^e^ Gleason score of 4 + 3 or greater or maximum cancer-core length greater than 50% with any PCa. The prevalence was 436 men (43% [95% CI, 40%-46%]).

^f^ Gleason score of 3 + 4 or greater. The prevalence was 475 men (47% [95% CI, 44%-50%]).

## Discussion

This study showed that a low-suspicion bpMRI had a high NPV in ruling out significant prostate cancer in confirmatory biopsies. The results suggest that bpMRI may be used as a triage test to exclude the presence of aggressive disease and avoid unnecessary biopsies with its inherent complications (severe infection, rectal bleeding, etc).^3,22^ Biparametric MRI suspicion scores were associated with prostate cancer detection rates, and performing combined biopsies (standard and targeted) in all men significantly enhanced the detection of significant prostate cancer compared with standard biopsies alone, which is the recommended diagnostic standard approach in biopsy-naive men. If combined biopsies were restricted solely to patients with suspicious bpMRIs, only 8 men with significant prostate cancer would have been missed and significantly fewer men (n = 77) with insignificant prostate cancer would have been diagnosed. Therefore, 305 of 1020 men (30%) could have safely avoided biopsies because most of these men had low-risk disease qualifying for surveillance. Reducing overdiagnoses of insignificant prostate cancer compared with standard biopsies (−40%) or combined biopsies in all men (−31%) might also reduce overtreatment.^2^

Our findings are consistent with those of the PROMIS study by Ahmed et al^7^ that provided level 1b evidence for the diagnostic accuracy of MRI in detecting prostate cancer. Those findings suggested that if mpMRI were used as a triage test, 1 in 4 men might safely avoid prostate biopsies and the diagnostic ratio of significant prostate cancer vs insignificant prostate cancer would be improved. In addition, the results from the PROTECT study by Hamdy et al^23^ were a critical landmark in showing that overdiagnosis and overtreatment of low-risk disease, resulting from the standard biopsy approach, showed minimal patient survival benefits, although it did decrease metastasis rates. The fact that more than three-fourths of the included patients had low-risk disease (the rest mostly intermediate risk) emphasizes the need to avoid biopsies and overtreatment of men.

Prior studies evaluated the diagnostic accuracy of bpMRI alone,^24^ combined with PSA levels,^25^ or compared with mpMRI.^26^ In a recent study of 161 biopsy-naive men who underwent bpMRI followed by targeted and standard biopsies, Jambor et al^24^ found that restricting biopsies to men with equivocal to highly suspicious bpMRI findings reduced the number of men undergoing biopsies by 24%, while failing to detect only 2% with significant prostate cancer. Although their results are similar to ours, Jambor and colleagues used a different bpMRI scoring system, they relied on cognitive targeted biopsies, and their biopsy operator was not blinded to the bpMRI findings before performing standard biopsies.

At present, the US Preventive Services Task Force recommends that PSA screening should be based on shared decision making and patient preferences for men aged 55 to 69 years. However, opponents of screening argue that the test has no net benefit and the harms (eg, high false-positive rate, overdetection of insignificant prostate cancer, and biopsy complications) outweigh the benefits demonstrated in randomized clinical trials.^27,28,29^ However, using MRI as a secondary triage test in men with elevated PSA levels could potentially minimize uncertainties and improve the balance between benefits and harms by reducing the number of false-positive PSA results that would otherwise lead to unnecessary invasive biopsies. The net benefit and decision curve analyses in our study showed that restricting biopsies to men with suspicious (modified PI-RADS 3-5) bpMRI lesions achieved the highest clinical value for all threshold probabilities compared with our current practice—standard biopsies in all men. However, at very low biopsy threshold probabilities, the preferable approach is to perform combined biopsies in all men. Assuming that no urologist would routinely carry out a biopsy in a man with less than a 5% risk of significant prostate cancer (equivalent to performing biopsies in 20 men to find 1 additional significant prostate cancer), using bpMRI to determine whether to perform a biopsy achieved the best clinical outcome balancing benefits and harms.

In general, we should cautiously consider using bpMRI or mpMRI as a triage test to identify individuals who can avoid prostate biopsies. Numerous factors, including image quality, interpretation, and definition of significant prostate cancer including disease prevalence, can affect the performance of targeted biopsies and the NPV of an MRI. A recent meta-analysis found that the median mpMRI NPVs (suspicion score ≥3) for ruling out any prostate cancer and significant prostate cancer were 82% and 88%, respectively. However, these values were strongly influenced by disease prevalence in the populations studied.^13^ We found a lower NPV for any prostate cancer (72%) for a modified PI-RADS score of 3 or higher, but a higher NPV for significant prostate cancer (97%), although the definitions of significant prostate cancer differed. Performing MRI can be expensive and time-consuming, and it would be a major challenge for any health care system to systematically use mpMRIs to diagnose prostate cancer before all biopsies. However, our results confirm that a more rapid and simple bpMRI approach is feasible, is sufficient for MRI/TRUS image fusion, and provides an accurate sector map of the prostate for targeted biopsies. It improves prostate cancer detection and risk stratification in biopsy-naive men and maintains the high diagnostic accuracy of mpMRI.^30,31^ Kuhl et al^26^ found no significant differences in the diagnostic accuracy of bpMRI and mpMRI in 542 men with elevated PSA who underwent repeated biopsies. However, it is important to note that a low-suspicion bpMRI did not unequivocally rule out any prostate cancer. Nevertheless, the key concern in clinical practice is to detect and rule out significant disease while avoiding unnecessary biopsies.

### Limitations

Our study had limitations. It was performed at a single center with 1 dedicated MRI physician reading the bpMRIs and 2 highly experienced TRUS operators performing biopsies. As a result, no interreader variability analyses were done. Less experienced readers and operators might not achieve the same diagnostic yield. Further work would be necessary to evaluate variability between experts and nonexperts. Second, all the patients in our study were from a non-PSA-screened population in whom benign reasons for elevated PSA levels (eg, urinary retention, urinary tract infections) had been ruled out before inclusion. This might explain both the rather high prostate cancer detection rate using standard biopsies and the higher median PSA level (8.0 ng/mL) compared with other studies.^7,11,12,24^ The diagnostic accuracy and NPVs of bpMRIs might be different in other patient populations. Third, we used biopsy results comparatively in this study, and the combined biopsy results from all patients were used as the reference standard. There may have been undetected prostate cancer lesions in both standard and targeted biopsy procedures, and the true rate of false-negative readings cannot be assessed. Nevertheless, we performed standard biopsies on all study participants, including those with low-suspicion bpMRI findings. This enabled us to compare outcomes among the different biopsy techniques and make comparisons that reflect clinical practice. Finally, the criteria for significant prostate cancer diagnoses depended on the histopathological assessment of biopsies. Although our definition is similar to that used in the PROMIS study by Ahmed et al,^7^ other investigators have used and suggested different definitions that might change the overall diagnostic accuracy of bpMRIs. A clear consensus for defining significant prostate cancer in MRI biopsy studies will be required to allow interstudy comparisons and to develop redefined risk calculators that include biopsy results from additional MRI targeted biopsies, as most of the currently available predictive nomograms and risk calculators are based on standard biopsy results with the inherent limitations of standard biopsy.

Despite these limitations, our data provide evidence for the reliability of using low-suspicion bpMRI findings as a noninvasive diagnostic tool to rule out more aggressive prostate cancer and avoid unnecessary biopsies. Although the use of prebiopsy bpMRI and targeted biopsies significantly improve risk stratification and could benefit clinical practice, the cost-effectiveness and long-term health outcomes using MRI have not been fully explored. Follow-up data and the long-term outcomes of these study patients will be assessed in the future. Furthermore, because bpMRI is a new diagnostic imaging approach, further studies are needed to validate our findings and fully explore the role of bpMRI in prostate cancer management before more widespread implementation into clinical practice.

## Conclusions

Low-suspicion bpMRI has a high NPV in ruling out significant disease in biopsy-naive men with clinical suspicion of prostate cancer. Furthermore, bpMRI suspicion scores are strongly associated with prostate cancer detection rates and performing biopsies (standard plus targeted) only in men with suspicious bpMRI findings is the preferred approach for improving the diagnostic ratio of significant prostate cancer to insignificant prostate cancer compared with our current diagnostic standard—standard biopsies in all men. Therefore, bpMRI used as a triage test improves risk stratification and allows for 30% of men with clinical suspicion of prostate cancer to safely avoid unnecessary prostate biopsies with their inherent risks. Further studies are needed to fully explore its future role in clinical prostate cancer management.

## Notes

Supplement. **eTable 1.** Biparametric MRI Sequence Parameters **eTable 2.** Patient Characteristics of Men (n = 8) With Low-Suspicion bpMRIs (PI_mod_ 1-2) **eFigure 1.** Comparison of bpMRI Suspicion Scores to Cancer Significance **eFigure 2.** Decision Curve Analysis
