# Supplementary material for: Assessment of the Diagnostic Accuracy of Biparametric Magnetic Resonance Imaging for Prostate Cancer in Biopsy-Naive Men: The Biparametric MRI for Detection of Prostate Cancer (BIDOC) Study
Source: JAMA Netw Open. 2018 Jun 8;1(2):e180219. doi: 10.1001/jamanetworkopen.2018.0219 (PMC6324414; doi:10.1001/jamanetworkopen.2018.0219)
Supplement: Supplement. — eTable 1. Biparametric MRI Sequence Parameters eTable 2. Patient Characteristics of Men (n = 8) With Low-Suspicion bpMRIs (PImod 1-2) eFigure 1. Comparison of bpMRI Suspicion Scores to Cancer Significance eFigure 2. Decision Curve Analysis [file jamanetwopen-1-e180219-s001.pdf]

Supplementary Online Content

Boesen L, Nørgaard N, Løgager V, et al. Assessment of the diagnostic accuracy of biparametric magnetic resonance imaging for prostate cancer in biopsy-naive men: the Biparametric MRI for Detection of Prostate Cancer (BIDOC) study. *JAMA Netw Open*. 2018;1(1):e180219. doi:10.1001/jamanetworkopen.2018.0219

eTable 1. Biparametric MRI Sequence Parameters

eTable 2. Patient Characteristics of Men (n = 8) With Low-Suspicion bpMRIs (PI_{mod} 1-2)

eFigure 1. Comparison of bpMRI Suspicion Scores to Cancer Significance

eFigure 2. Decision Curve Analysis

This supplementary material has been provided by the authors to give readers additional information about their work.

© 2018 Boesen L et al. *JAMA Network Open*.

eTable 1. Biparametric MRI Sequence Parameters

Sequence	TR (ms)	TE (ms)	NEX/Averages	FOV (mm)	Resolution/ Voxel size (mm)	Slices (n)	Slice thickness (mm)	Gap (mm)	Time (min:sec)
Sagittal luxury scout	3.3	1.65	2	AP 270<br>LR 55<br>FH 270	ACQ:<br>1.5 × 1.5 × 3<br>Recon:<br>0.84 × 0.84 × 3	14	3	1	00:29
T2W TSE Axial	3,745	90	1	AP 180<br>LR 180<br>FH 104	ACQ:<br>0.45 × 0.45 × 3<br>Recon:<br>0.22 × 0.22 × 3	30	3	0.5	8:52
Diffusion EPI b-value: 0, 100, 800, 2,000	9,983	71	2	AP 180<br>LR 180<br>FH 104	ACQ:<br>2.1 × 2.2 × 4<br>Recon:<br>0.8 × 0.8 × 4	26	4	0	6:30

T2W = T2-weighted imaging; TSE = turbo spin echo; EPI = echo planar imaging; TR = repetition time; TE = echo time; FA = flip angle; ACQ = acquisition matrix; Recon = reconstruction; NEX = number of excitations/average signals; FOV = field of view; AP = anterior–posterior; LR = left–right; FH = foot–head

© 2018 Boesen L et al. *JAMA Network Open*.

eTable 2. Patient Characteristics of Men (n = 8) With Low-Suspicion bpMRIs (PI_{mod} 1-2)

Patient ID	Time MRI to bx (days)	Age (years)	PSA (ng/ml)	cT_{DRE} stage	Prostate volume (ml)	PSA density (ng/ml/cc)	Standard PCa positive cores (num/10)	Gleason score / Grade group	MCCL	BpMRI PI_{mod} score
420	7	67	4.4	T1c	37	0.12	2	4+3 / 3	25	2
536	9	70	7.2	T2c	54	0.13	10	4+5 / 5	100	1
582	2	54	4.7	T1c	34	0.14	1	4+4 / 4	10	1
649	6	62	5.9	T1c	58	0.10	2	4+3 / 3	35	2
701	2	57	14	T1c	81	0.17	4	3+4 / 2	70	1
839	6	70	8.7	T1c	48	0.18	2	4+3 / 3	50	1
911	6	72	4.7	T2b	18	0.26	5	4+3 / 3	85	1
1013	2	66	5.4	Tx	88	0.06	1	4+4 / 4	15	1
Median [IQR]	6 [6–6]	67 [67–70]	5.7 [5.7–7.6]		51 [51–64]	0.14 [0.14–0.17]			43 [43–74]	

PCa = prostate cancer; cT_{DRE} = tumor stage by digital rectal examination; MCCL = maximum cancer-core length; IQR = Inter quartile range; PI_{mod} = modified prostate imaging reporting and data system score

© 2018 Boesen L et al. *JAMA Network Open*.

eFigure 1. Comparison of bpMRI Suspicion Scores to Cancer Significance

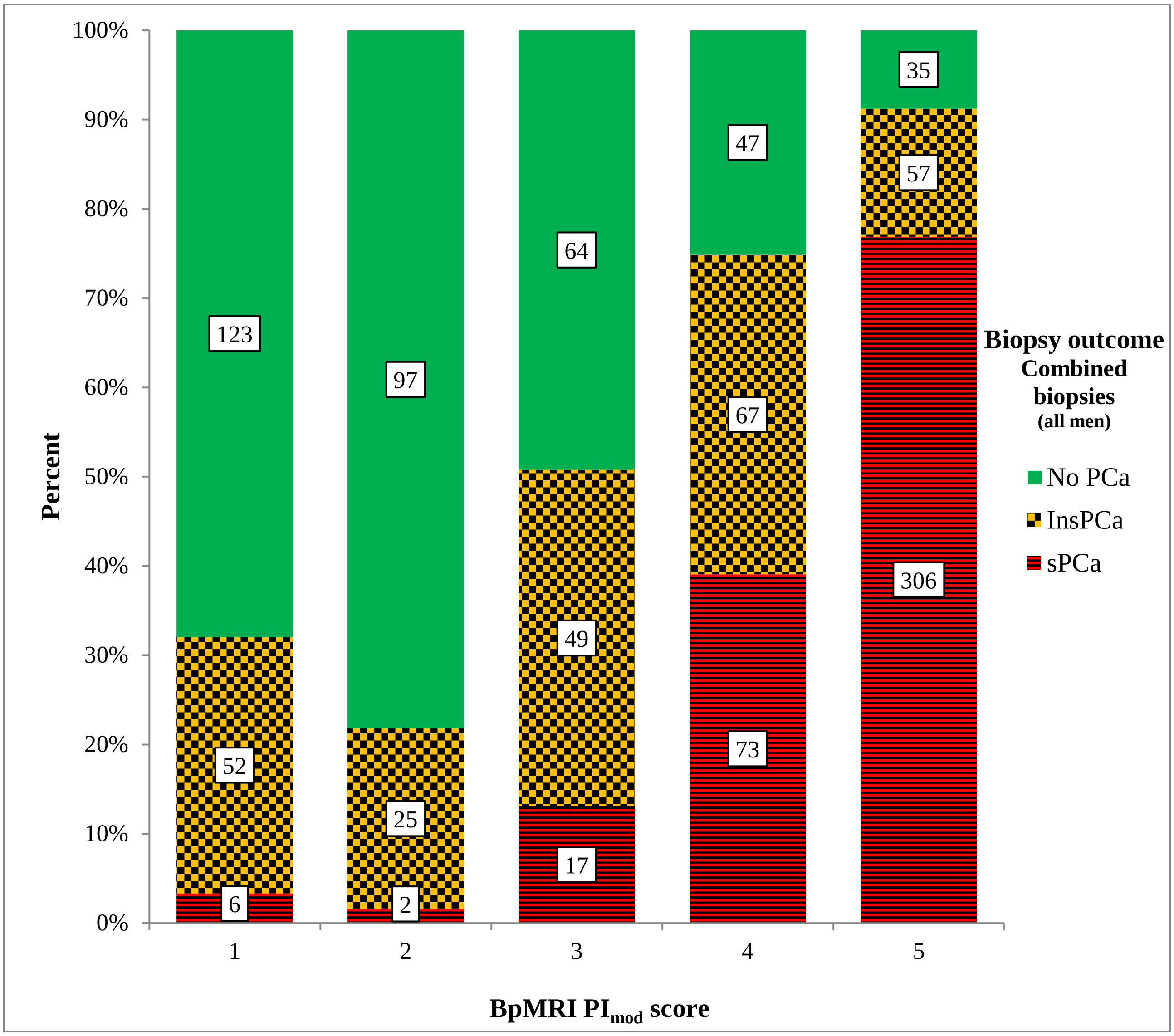

PCa = prostate cancer; InsPCa = insignificant PCa; sPCa = significant PCa; bpMRI = biparametric MRI; PI_{mod} = modified prostate imaging reporting and data system

Prostate cancer detection rates for all patients (N = 1020) based on combined (standard plus targeted) biopsy results and stratified by cancer significance (primary definition) within each bpMRI PI_{mod} score. All men underwent standard biopsies, but only those with bpMRI PI_{mod} 3–5 also underwent targeted biopsies.

© 2018 Boesen L et al. *JAMA Network Open*.

eFigure 2. Decision Curve Analysis

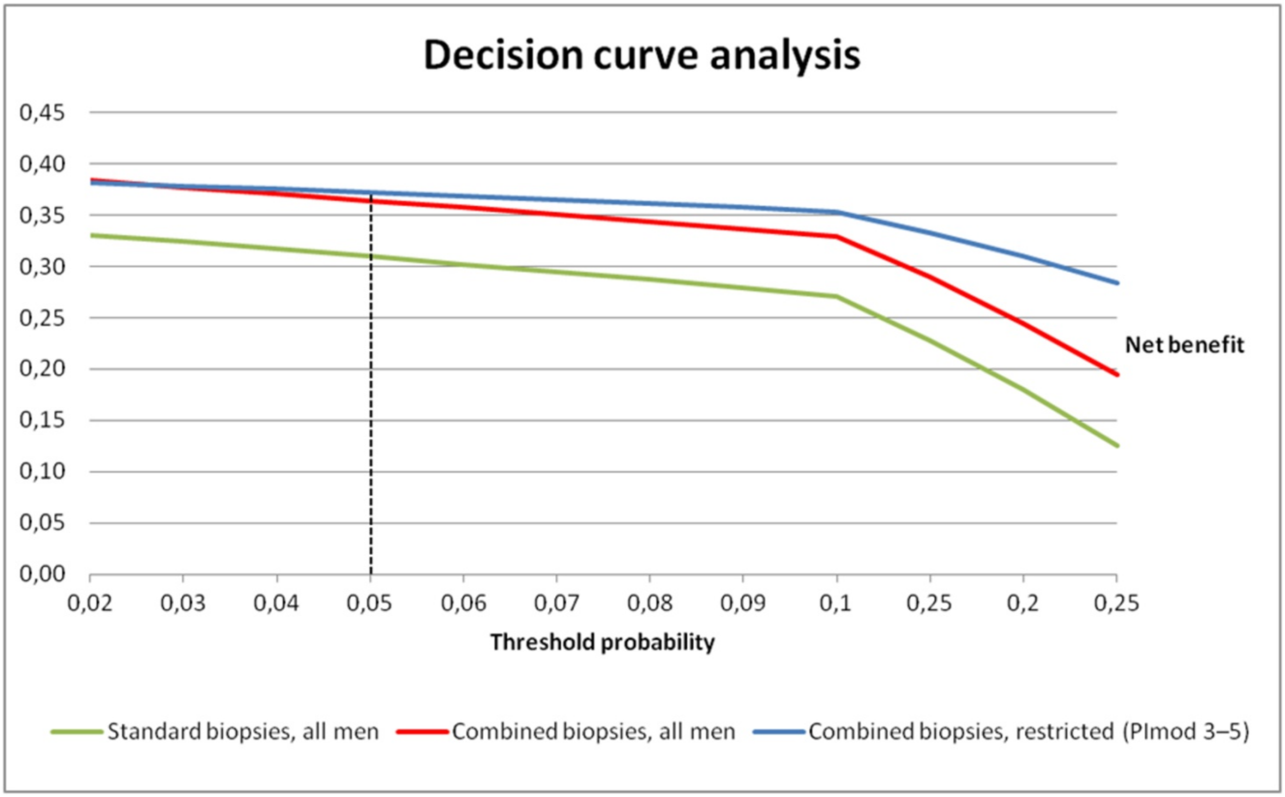

Decision curve showing net benefit for carrying out biopsies in men at risk of significant prostate cancer. The net benefit for carrying out standard biopsies in all men (standard approach) is lower for all threshold probabilities compared to using bpMRI as a triage test and perform combined biopsies restricted to men with suspicious bpMRI (PI_{mod} 3–5). The dotted vertical line indicates a threshold of 5 % equivalent to perform biopsies in 20 men to find one additional sPCa.

© 2018 Boesen L et al. *JAMA Network Open*.
